# Effects of Ursolic Acid on Immune Function and Antioxidative Capacity in Weaned Rabbits

**DOI:** 10.3390/ani15152159

**Published:** 2025-07-22

**Authors:** Yanhua Liu, Saijuan Chen, Fengyang Wu, Baojiang Chen, Chong Li, Xinyu Yang, Gang Zhang, Man Hu

**Affiliations:** 1 College of Veterinary Medicine, Hebei Agricultural University, Baoding 071000, China; liuyanhuagood@126.com; 2 College of Animal Science and Technology, Hebei Agricultural University, Baoding 071000, China; chensaijuan@126.com (S.C.); chenbaojiang@vip.sina.com (B.C.); masterli1989@sina.com (C.L.); yangxyily@outlook.com (X.Y.); 3 College of Food Science and Technology, Hebei Agricultural University, Baoding 071000, China; fengyangwu2020@163.com

**Keywords:** ursolic acid, weaned rabbits, growth performance, antioxidant, anti-inflammatory

## Abstract

This study aimed to investigate the effects of dietary supplementation with different levels of ursolic acid (UA) on the growth performance, immune function, intestinal antioxidant capacity, and anti-inflammatory responses of weaned rabbits. A total of 160 Hyla meat rabbits aged 35 days were randomly assigned to four groups. Each treatment group consisted of 8 replicates, with 5 rabbits per replicate. The rabbits were fed a basal diet (control group, CON) or experimental diets supplemented with 50, 100, or 200 mg/kg UA for 28 days. Dietary supplementation with 50 mg/kg UA significantly increased (*p* < 0.05) the average daily gain and average daily feed intake. The villus height, crypt depth, and villus height to crypt depth ratio exhibited quadratic responses (*p* < 0.05) to increasing dietary UA levels, with rabbits fed 50 mg/kg UA showing optimal ileal morphology. Compared with the CON group, dietary supplementation with 50 mg/kg UA significantly enhanced (*p* < 0.05) cecal catalase activity, secretory immunoglobulin A, and interleukin-10 (IL-10) levels, while the addition of 200 mg/kg UA increased (*p* < 0.05) serum catalase activity. The concentrations of serum tumor necrosis factor-α (TNF-α) and cecal IL-10 responded quadratically (*p* < 0.01 and *p* = 0.01, respectively) as the dietary UA level increased. With increasing UA supplementation, cecal Kelch-like ECH-associated protein 1 and *IL-10* mRNA expression showed linear upregulation (*p* < 0.05), whereas nuclear factor erythroid 2-related factor 2 (*Nrf2*), superoxide dismutase 1 (*SOD1*), quinone oxidoreductase 1 (*NQO1*), TNF-α, interleukin-6, and interleukin-8 displayed quadratic responses (*p* < 0.05). Dietary UA at 50 mg/kg significantly downregulated cecal *TNF-α* and interleukin-1β mRNA expression while upregulating *Nrf2*, *NQO1*, and *SOD1* mRNA levels (*p* < 0.05). In conclusion, dietary supplementation with 50 mg/kg UA significantly improved the growth performance of weaned rabbits by improving intestinal morphology, immune function, and antioxidant and anti-inflammatory capacities, demonstrating its efficacy as a natural phytogenic feed additive.

## Simple Summary

In intensive livestock production systems, oxidative stress and intestinal inflammation are critical limiting factors affecting animal growth performance. Ursolic acid (UA), a natural pentacyclic triterpenoid compound, exhibits significant biological activities such as antioxidant and anti-inflammatory effects. This study used different levels of UA supplementation in basal diets to evaluate its effects on the growth performance, immune function, and antioxidant capacity of weaned rabbits. The findings revealed that UA supplementation significantly enhanced growth performance, improved systemic immune responses, optimized intestinal antioxidant status, and effectively mitigated inflammatory reactions. These results substantiate the potential of UA as a novel phytogenic feed additive for alleviating oxidative stress and intestinal inflammation in intensively reared meat rabbits, highlighting its significant application value in modern animal production systems.

## 1. Introduction

Rabbit meat is increasingly gaining consumer recognition and preference due to its delicate flavor and superior nutritional profile, characterized by a low fat content, minimal cholesterol levels, and abundant levels of highly digestible proteins and unsaturated fatty acids [1,2]. In recent years, global rabbit meat production has shown significant growth, with China becoming the world’s leading producer and consumer [2,3]. In meat rabbit production, intestinal health management is particularly crucial as it directly affects growth performance and immune function. This is especially true during the weaning period, when rabbits are susceptible to multiple stressors due to their immature intestinal barrier function, including oxidative stress and pathogenic bacterial infections [4,5]. These factors often lead to severe consequences such as diarrhea, growth retardation, and even mortality, causing significant economic losses in the industry [6,7]. Over the past few decades, antibiotics have been widely used as growth promoters in weaned rabbits [8]. While they have indeed helped alleviate weaning stress and improve production efficiency to some extent, the overuse of antibiotics has led to increasingly severe issues, including bacterial resistance and drug residues in animal products, posing significant potential risks to human health [5]. Therefore, it is necessary to develop safe and efficient nutritional regulation alternatives to improve intestinal health and enhance immune function in weaned rabbits.

Ursolic acid (UA), a naturally pentacyclic triterpenoid compound widely found in fruits, edible plants, and medicinal herbs, has garnered significant attention due to its notable anti-inflammatory, antioxidant properties and diverse biological effects [9,10,11]. In recent years, UA has demonstrated considerable application value in feed additives, functional food development, and nutritional interventions for metabolic syndromes and oxidative-stress-related diseases. Dietary UA supplementation has been shown to improve broiler growth performance by elevating serum and jejunal antioxidant capacity, attenuating intestinal inflammation, and modulating cecal microbial composition [12]. Wang et al. [13] also found that UA not only improved the growth performance and intestinal antioxidant status of largemouth bass but also enhanced intestinal barrier function through the specific modulation of bacterial abundances, particularly Tenericutes and Firmicutes. Similarly, murine studies further demonstrated that UA supplementation effectively improves intestinal immune homeostasis, enhances gut barrier function, and consequently promotes overall intestinal health [11,14]. Furthermore, UA could alleviate colitis in mice by suppressing inflammatory responses in intestinal epithelial cells and macrophages through the modulation of the nuclear factor-kappa B (NF-κB) signaling pathway [15].

However, research on the application of UA in rabbit production remains scarce. Given that weaned rabbits are highly susceptible to oxidative stress and intestinal inflammation, leading to growth impairment, investigating the regulatory effects of UA on weaned rabbits holds significant importance. This study aims to investigate the effects of dietary supplementation with different levels of UA on the growth performance, immune function, as well as intestinal anti-inflammatory capacity and antioxidant capacity of weaned rabbits, providing a scientific basis for UA as a natural feed additive in the healthy breeding of rabbits.

## 2. Materials and Methods

The feeding trial was conducted at the Animal Husbandry Experiment Base of Hebei Agricultural University. The experiment was approved by the Animal Ethics Committee of Hebei Agricultural University (approval No.: 2024162). Ursolic acid was provided by Hebei Chenguang Bio-Technology Co., Ltd. (Handan, China), with a guaranteed purity of ≥90%.

### 2.1. Experimental Design, Animals, and Management

A total of 160 healthy 35-day-old weaned Hyla meat rabbits, with an initial average weight of 0.84 ± 0.09 kg and an equal number of males and females, were selected and randomly allocated into four treatment groups. Each treatment group consisted of 8 replicates, with 5 rabbits per replicate. The control group was fed a basal diet, while the experimental groups were fed the basal diet supplemented with 50, 100, or 200 mg/kg of UA. The pre-trial period lasted 7 days, followed by a formal trial period of 28 days. During the pre-trial period, all rabbits were fed the basal diet. The basal diet was formulated in accordance with the Nutrient Requirements for Meat Rabbits (NY/T 4049-2021, Ministry of Agriculture of China) [16]. The specific composition and nutritional levels of the basal diet are presented in Table 1.

The rabbits were housed in a three-tiered stepped cage system, with individual cage dimensions of 120 × 80 × 60 cm. All rabbits were housed in a temperature-controlled environment at 22 °C ± 2 °C with 50–60% relative humidity under a 14:10 h light:dark cycle. All rabbits were provided ad libitum access to water and feed. Immunization was performed according to the standard protocol. Prior to and throughout the experiment, rabbit housing, feeding, and drinking facilities were rigorously disinfected, and regular maintenance to ensure hygiene in the rabbit houses was performed.

### 2.2. Sample Collection

Initial (42 days of age) and final (70 days of age) body weights (BWs) were determined for all rabbits in each group after a 12 h fasting period (with free access to water) at the beginning and end of the formal trial. The given feed and leftover feed weight for each replicate were recorded throughout the experiment. The average daily feed intake (ADFI), average daily gain (ADG), and feed to gain ratio (F/G) were calculated. Fecal consistency was scored on a 4-point scale (0, normal; 1, soft feces stools; 2, soft/liquid stools; 3, liquid stools) by two blinded, trained assessors who were unaware of dietary treatment allocations. Diarrhea was defined as a fecal consistency score ≥2. Additionally, the diarrhea rate for each group was recorded as:

Diarrhea rate (%) = [∑(number of rabbits with diarrhea × number of days with diarrhea)/(total number of rabbits × number of experimental days)] × 100(1)

A representative sample of 500 g of feed was collected and ground through a 40-mesh sieve prior to the start of the experiment. The sample was analyzed for dry matter (DM; method 930.15), crude protein (CP; method 984.13), ether extract (EE; method 920.39), crude fiber (CF, method 978.10), Ca (method 968.08), and total P (method 965.17) in accordance with AOAC procedures [17]. Neutral detergent fiber (NDF) and acid detergent fiber (ADF) were determined using a fiber analyzer (Ankom Technology, Macedon, NY, USA) following the methodology of van Soest et al. [18].

On d 35, one rabbit with a body weight closest to the group average was selected from each replicate (excluding blocks with the highest and lowest initial weights) for collection of 5 mL blood samples via ear vein puncture into vacuum container tubes (Becton Dickinson Vacutainer Systems, Franklin Lakes, NJ, USA). The blood samples were allowed to clot at room temperature for 30 min and then centrifuged at 3000 r/min for 10 min. The obtained serum was collected and stored at −20 °C until further analysis.

At the end of the experiment, the rabbits that provided blood samples were euthanized, after which the middle sections of the ileum and cecum were collected. After removing intestinal contents, the segments were rinsed with saline solution. A 2 cm ileal sample was fixed in 4% paraformaldehyde solution for histopathological analysis, while 4 cm cecal segments were immediately frozen in liquid nitrogen and then stored at −80 °C.

Immediately following euthanasia, the liver, kidney, and spleen were excised intact, and all attached connective tissues, fat, and other adherent tissues were carefully removed. The organs were then weighed after wiping off surface blood stains with filter paper. The organ index was calculated as the ratio of the organ weight to the final body weight.

### 2.3. Intestinal Histological Analysis

After fixation in 4% paraformaldehyde for 48 h, the ileal tissues were processed through ethanol dehydration, xylene clearing, and paraffin embedding. Sections were stained with H&E after standard dewaxing and rehydration. The sections were scanned using a panoramic slide scanner (Pannoramic DESK, 3DHISTECH, Budapest, Hungary) to obtain comprehensive images of ileum histological damage. Images at 50× and 200× magnifications were captured from selected observation areas. Villus height (VH) and crypt depth (CD) were measured using scanning software (CaseViewer 2.4, 3DHISTECH, Budapest, Hungary) and analysis software (Image-Pro Plus 6.0, Media Cybernetics, Rockville, MD, USA). The villus height to crypt depth ratio (VH/CD) was subsequently calculated.

### 2.4. Blood and Tissue Indicators

For tissue homogenate preparation, 0.1 g of cecal tissue was accurately weighed and mixed with 0.9 mL of pre-cooled physiological saline. The samples were ground at low temperature to prepare a 10% tissue homogenate. The homogenates were centrifuged at 4 °C and 5000 rpm for 10 min, and the supernatant was collected. ELISA kits provided by Beijing Borui Changyuan Technology Co., Ltd., Beijing, China, were used to determine the serum and cecal levels of immunoglobulin M (IgM), immunoglobulin G (IgG), immunoglobulin A (IgA), complement 3 (C3), complement 4 (C4), secretory immunoglobulin A (sIgA), tumor necrosis factor-α (TNF-α), interleukin-1β (IL-1β), interleukin-6 (IL-6), interleukin-8 (IL-8), interleukin-10 (IL-10), total antioxidant capacity (T-AOC), glutathione peroxidase (GSH-Px), superoxide dismutase (SOD), catalase (CAT), and malondialdehyde (MDA). All procedures followed the manufacturer’s instructions.

### 2.5. Cecum-Related Gene Expression

The mRNA expression levels of *TNF-α*, *IL-1β*, *IL-6*, *IL-8*, *IL-10*, Kelch-like ECH-associated protein 1 (*Keap1*), nuclear factor erythroid 2-related factor 2 (*Nrf2*), heme oxygenase 1 (*HO-1*), quinone oxidoreductase 1 (*NQO1*), superoxide dismutase 1 (*SOD1*), and glutathione peroxidase (*GSH-Px*) in the cecum were detected by quantitative real-time PCR (qRT-PCR). The primer sequences for quantitative real-time PCR are presented in Table 2. The total RNA was extracted from cecum tissues using a magnetic-bead-based method, and its concentration and purity were determined by measuring the absorbance at 260 nm and 280 nm. Reverse transcription was performed according to the manufacturer’s instructions (TaKaRa, Kusatsu, Japan), with the reaction conditions set at 37 °C for 10 min, followed by 85 °C for 5 s to obtain cDNA. The quantitative PCR amplification was conducted under the following conditions: pre-denaturation at 95 °C for 30 s; denaturation at 94 °C for 5 s, annealing at 60 °C for 10 s, for a total of 40 cycles. The relative gene expression levels were calculated using the 2^−ΔΔCt^ method.

### 2.6. Data Statistics and Analysis

The experimental data were analyzed by using one-way ANOVA with SPSS 22.0 and GraphPad Prism 6 software, and multiple comparison tests were performed using the LSD and Tukey’s methods. Polynomial contrasts assessed linear and quadratic trends in UA dose–response relationships. The diarrhea rate and mortality rate were compared with a chi-squared test. Statistical significance was defined as *p* < 0.05.

## 3. Results

### 3.1. Growth Performance and Diarrhea Rate

Compared with the CON group, dietary supplementation with 50 mg/kg of UA significantly increased (*p* < 0.05) the ADG and ADFI of the weaned rabbits (Table 3). However, there were no significant differences (*p* > 0.05) in initial BW, final BW, F/G, or diarrhea rate among the treatment groups. The final BW showed a quadratic response (*p* < 0.05) to increasing dietary UA supplementation levels. Furthermore, the mortality rates were 2.50%, 2.50%, 5.00%, and 5.00%, respectively, for the CON, UA50, UA100, and UA200 groups.

### 3.2. Organ Indices

As shown in Table 4, no significant differences (*p* > 0.05) were observed in the indices of the liver, kidney, and spleen among the groups.

### 3.3. Intestinal Morphology

In Table 5, the ileal morphology parameters, including villus height, crypt depth, villus height to crypt depth ratio, exhibit quadratic responses(*p* < 0.05) to increasing dietary UA levels. Compared to the CON group, the ileal villus height and villus height to crypt depth ratio of the weaned rabbits fed 50–200 mg/kg UA were significantly increased (*p* < 0.05). Additionally, the ileal crypt depth in weaned rabbits fed 50 mg/kg UA was significantly lower (*p* < 0.05) than that in the CON group.

### 3.4. Serum and Cecal Immune Capacity

Both the serum C4 concentration and cecal sIgA level changed quadratically (*p* = 0.03 and *p* < 0.01, respectively) as the dietary UA level increased (Table 6). Furthermore, the cecal sIgA level in the weaned rabbits fed 50 mg/kg UA was significantly higher (*p* < 0.05) than that in the CON group.

### 3.5. Serum and Cecum Antioxidant Indicators

Serum CAT activity exhibited a significant linear dose–response relationship to increasing UA supplementation (*p* = 0.01). Compared with the CON group, dietary supplementation with 200 mg/kg UA significantly enhanced (*p* < 0.05) serum CAT activity, while the addition of 50 mg/kg UA significantly increased (*p* < 0.05) cecal CAT activity (Table 7).

### 3.6. Serum and Cecal Inflammatory Cytokines

The concentrations of serum TNF-α and cecal IL-10 responded quadratically (*p* < 0.01 and *p* = 0.01, respectively) as the dietary UA level increased (Table 8). Dietary supplementation with 50–200 mg/kg UA significantly decreased (*p* < 0.01) the serum levels of both TNF-α and IL-8. Dietary supplementation with 50 mg/kg UA significantly increased (*p* < 0.05) the cecal IL-10 levels, while 200 mg/kg UA markedly reduced (*p* < 0.05) the cecal IL-1β levels compared to the CON group. Furthermore, dietary UA inclusion linearly decreased (*p* < 0.01) the serum IL-8 and cecal IL-1β concentrations.

### 3.7. Cecal-Antioxidant-Related Gene Expression

**Figure 1:**
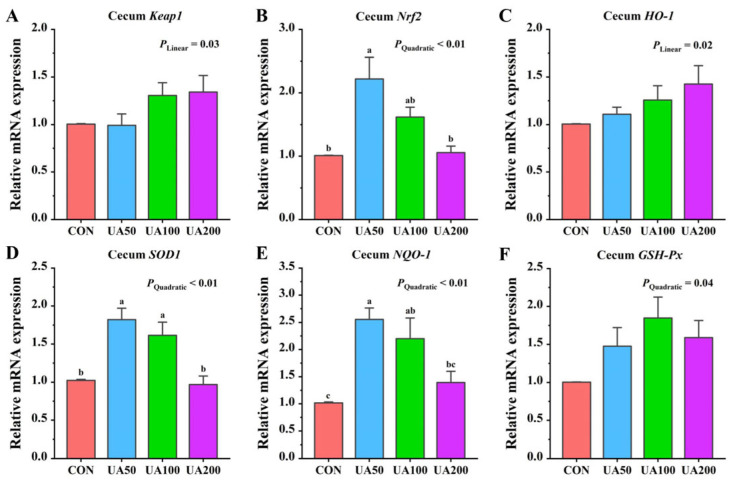
Effects of ursolic acid on expression levels of antioxidant-related genes in cecum of weaned rabbits. (**A**) *Keap1*; (**B**) *Nrf2*; (**C**) *HO-1*; (**D**) *SOD1*; (**E**) NQO-1; (**F**) *GSH-Px*. ^a–c^ Means with different letters differ significantly among treatments.

With increasing UA supplementation, the relative mRNA expression of cecal *Keap1* showed a significant linear upregulation (*p* < 0.05), whereas the relative expression levels of *Nrf2*, *SOD1*, and *NQO-1* mRNA displayed quadratic responses (*p* < 0.05, Figure 1). The relative expression level of *Nrf2* mRNA in the cecum of the 50 mg/kg UA group was significantly higher (*p* < 0.05) than that in the CON group. Both the 50 mg/kg and 100 mg/kg UA groups showed significantly higher (*p* < 0.05) relative mRNA expression levels of *NQO-1* and *SOD1* in the cecum compared to the CON group.

### 3.8. Cecal Inflammation-Related Gene Expression

**Figure 2:**
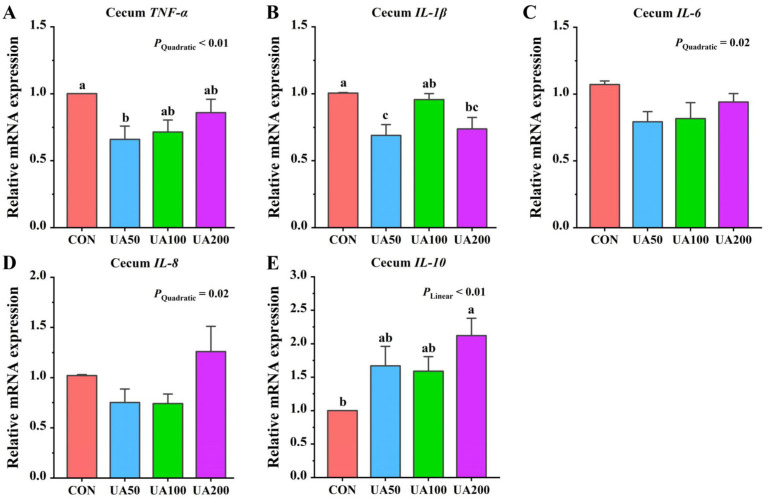
Effects of ursolic acid on expression levels of inflammation-related genes in cecum of weaned rabbits. (**A**) *TNF-α*; (**B**) *IL-1β*; (**C**) *IL-6*; (**D**) *IL-8*; (**E**) *IL-10*. ^a–c^ Means with different letters differ significantly among treatments.

With increasing UA supplementation, the relative mRNA expression of cecal *IL-10* showed a linear increase (*p* < 0.01), while *TNF-α*, *IL-6*, and *IL-8* exhibited quadratic responses (*p* < 0.05, Figure 2). Compared to the CON group, the 50 mg/kg UA group showed lower (*p* < 0.05) relative expression of *TNF-α* in the cecum, while the 200 mg/kg UA group exhibited higher (*p* < 0.05) *IL-10* expression in the cecum (Figure 2). Both the 50 mg/kg and 200 mg/kg UA groups showed significantly lower (*p* < 0.05) relative expression of *IL-1β* mRNA in the cecum compared to the CON group.

## 4. Discussion

The plant-derived compound UA has a wide range of plant sources and high biosafety [10]. It can regulate the antioxidant and anti-inflammatory responses in animals through multiple targets and pathways, improving glucose and lipid metabolism levels, thereby promoting animal health and improving growth performance [11,19,20]. In the present study, we found that dietary supplementation with 50 mg/kg UA significantly increased both the ADG and ADFI in the weaned rabbits. These results align with the existing literature documenting the growth-promoting properties of UA in various species. Specifically, UA supplementation has been shown to markedly increase body weight gain and ADFI in mice [11,21]. Zhang et al. [12] also reported that dietary UA supplementation significantly improved ADG in broilers. Comparable growth-enhancing effects of UA, including increased ADG and improved feed efficiency, have also been documented in aquatic species such as largemouth bass (*Micropterus salmoides*) and gilthead seabream (*Sparus aurata*) [13,22]. Furthermore, dietary UA supplementation at doses ranging from 50 to 200 mg/kg showed no significant effects on organ indices, demonstrating its safety within this concentration range, without negative impacts on metabolism or immune organ development in rabbits. Zhang et al. [12] found that a higher UA dosage of 450 mg/kg effectively optimized intestinal morphology and improved growth performance in broilers. Despite species differences, the demonstrated efficacy of this higher dose in broilers provides supportive evidence for the safety of UA application in rabbits.

The intestine is the primary site for nutrient digestion and absorption, and its structural integrity is critical for maintaining barrier function. However, weaning stress often impairs intestinal barrier function and morphology in rabbits [23]. In this study, dietary UA supplementation alleviated weaning-stress-induced intestinal morphological damage. This aligns with studies in mice and broilers, where UA increased the jejunal villus height and the villus height to crypt depth ratio while reducing the crypt depth [11,12,21]. Ursolic acid may improve intestinal morphology by modulating gut microbiota composition and reducing pathogenic bacterial load [11]. The upregulatory effect of UA on tight junction protein expression also contributes to improved intestinal morphology [11,21], though future studies are needed to verify this at the molecular level. Moreover, a higher villus height to crypt depth ratio generally indicates improved intestinal development and enhanced nutrient absorption capacity [23], potentially explaining the UA-mediated growth promotion observed in the weaned rabbits.

Immunoglobulins reflect the capacity of antibody-mediated immune responses, and their serum concentrations serve as indicators for assessing immune function [24]. The complement system, a vital part of innate immunity, mediates immune regulation and host defense via cascade activation [25]. Although the serum C4 concentration exhibited a quadratic response to increasing dietary UA levels, supplementation with 50–200 mg/kg UA did not induce statistically significant alterations in C4 concentrations. This observation suggests that UA supplementation preserves systemic immune homeostasis, supporting its safety profile for potential long-term or high-dose applications. Furthermore, secretory IgA, a pivotal effector molecule in mucosal immunity, was significantly elevated in the UA-treated group. This increase suggests that UA enhances local mucosal immune responses, thereby improving resistance to pathogen adhesion and promoting intestinal mucosal homeostasis [26,27]. The increased cecal sIgA may enhance the epithelial mucosal barrier, which could be one of the factors contributing to the higher villus height observed in the UA supplemented group.

Antioxidant enzymes such as SOD, GSH-Px, and CAT are recognized as the primary defense against oxygen free radicals and superoxides [28]. The total antioxidant capacity reflects the synergistic effects of both the enzymatic and non-enzymatic antioxidant systems [29]. Together, these coordinated defense mechanisms play a crucial role in maintaining the dynamic redox balance within the organism. In this study, serum CAT activity exhibited a linear increase with rising dietary UA levels. Moreover, supplementation with 50 mg/kg UA enhanced the CAT activity in the cecum. These findings partially support our meta-analysis results showing UA’s ability to improve antioxidant enzyme activities and alleviate oxidative stress in animal tissues [30]. The antioxidant efficacy of UA appears particularly pronounced under pathological conditions or oxidative stress. Compared to healthy states, cells and animals experiencing inflammation or oxidative damage may benefit more from UA supplementation [30]. This is supported by evidence that UA effectively mitigates reactive oxygen species (ROS) accumulation in oxidatively injured cells and restores SOD activity [31,32]. The mechanism behind these effects appears multifaceted. UA enhances the endogenous antioxidant enzyme system to scavenge excessive ROS, thereby reducing lipid peroxidation damage. Previous studies have confirmed that UA also activates the AMP-activated protein kinase (AMPK) signaling pathway, thereby downregulating the expression of NADPH oxidases (such as NOX4) and reducing ROS production at its source [33,34]. Moreover, AMPK activation can further alleviate oxidative stress by improving mitochondrial function and suppressing inflammatory responses [35]. However, this study did not determine the effects of UA on AMPK expression levels, which is a limitation that warrants further investigation.

The nuclear factor Nrf2 is a key regulator of cellular antioxidant stress responses and plays a crucial regulatory role in various tissues [36]. Studies have shown that UA can exert antioxidant effects by modulating the Nrf2 signaling pathway [10,37]. For instance, in liver protection, research demonstrated that UA alleviated carbon-tetrachloride-induced hepatic oxidative stress in mice by promoting Nrf2 nuclear translocation and upregulating the expression of antioxidant proteins, including HO-1 and NQO-1 [38]. It has also been found that UA can alleviate calcium-oxalate-induced oxidative damage in mouse renal tubular epithelial cells by regulating the Nrf2/HO-1 signaling pathway, significantly increasing SOD activity while decreasing MDA levels [32]. To further validate this mechanism, Ding et al. [39] conducted comparative studies using wild-type and *Nrf2* knockout mice. Their results clearly demonstrate that UA’s neuroprotective effects depend on Nrf2 activation and the subsequent upregulation of downstream antioxidant proteins. At the molecular level, UA regulates Nrf2 expression primarily through epigenetic mechanisms involving DNA methylation and histone modifications, which reduce repressive modifications in the Nrf2 promoter region, thereby enhancing its transcription [40]. In the present study, we found that dietary UA supplementation increased the relative mRNA expression of *Nrf2*, *NQO-1*, and *SOD1* in the cecum. These findings strongly support the existing literature on the Nrf2 pathway’s role in antioxidant defense, further supporting the importance of Nrf2 signaling in UA-mediated antioxidant effects.

In our study, UA at doses of 50–200 mg/kg significantly reduced the serum levels of TNF-α and IL-8, while 50 mg/kg UA markedly increased cecal IL-10 levels. These findings are consistent with numerous in vivo and in vitro studies demonstrating UA’s ability to suppress pro-inflammatory cytokines such as IL-1β, IL-6, and TNF-α [11,13,32]. Specifically, research in broilers revealed that UA decreased *IL-6* gene expression in the jejunum and ileal mucosa and reduced the serum IL-6 and IL-1β levels [12]. The compound’s anti-inflammatory properties have been further validated in various disease models. In DSS-induced colitis, UA lowered IL-6 levels and attenuated inflammatory cell infiltration [14], while in LPS-induced lung injury, it suppressed TNF-α, IL-1β, and IL-6 production while upregulating anti-inflammatory IL-10 expression [41]. UA achieves these effects primarily through the immunomodulation of T-cell differentiation by promoting regulatory - cell (Treg) development and simultaneously inhibiting Th17 cell activation [11,42]. Since Tregs serve as major IL-10 producers, their UA-induced expansion leads to elevated IL-10 levels, which in turn suppress macrophage and neutrophil activation through negative feedback regulation, ultimately reducing the production of various pro-inflammatory cytokine [15]. These findings collectively highlight UA’s multifaceted anti-inflammatory action through cytokine modulation and immune cell regulation.

NF-κB is a key transcriptional regulator of multiple pro-inflammatory genes, whose aberrant activation in *Nrf2*-knockout mice underscores Nrf2’s vital role in inflammatory regulation [37]. Through the upregulation of anti-inflammatory enzymes including HO-1 [38], Nrf2 suppresses the production of various pro-inflammatory mediators such as cytokines, chemokines, adhesion molecules, and inducible nitric oxide synthase [38]. Furthermore, the Nrf2/HO-1 signaling pathway also can shift macrophage polarization toward the M2 anti-inflammatory phenotype, thereby decreasing pro-inflammatory factor release [43]. The observed elevation in the *Nrf2* mRNA expression in the cecal tissues, concomitant with the decreased *TNF-α* and *IL-1β* expressions, provides partial evidence that UA may exert anti-inflammatory effects via the Nrf2 pathway. Since excessive ROS can activate the NF-κB inflammatory signaling pathway and induce apoptosis, UA may also directly inhibit inflammation by decreasing ROS production [32]. Research has demonstrated that the anti-inflammatory mechanism of UA also involves the effective modulation of the NF-κB signaling pathway [44]. Ursolic acid inhibits IκBα degradation, thereby suppressing NF-κB nuclear translocation and its DNA-binding activity, ultimately leading to the significant downregulation of downstream inflammatory mediators such as TNF-α and IL-6 at the transcriptional level [45]. It further enhances this anti-inflammatory effect by suppressing NF-κB phosphorylation, which consequently blocks the activation of the downstream NLR family pyrin domain-containing 3 (NLRP3) inflammasome [44]. These molecular insights corroborate our previous transcriptomic data demonstrating UA’s suppressive effects on both NF-κB and NOD-like receptor signaling pathways [11], collectively highlighting its multifaceted anti-inflammatory properties.

## 5. Conclusions

In conclusion, UA significantly enhances the growth performance of weaned rabbits by improving intestinal morphology, boosting immune function, and strengthening antioxidant and anti-inflammatory capacities. As a highly promising plant-derived compound, UA demonstrates remarkable anti-inflammatory and antioxidant efficacy. Among the tested doses, 50 mg/kg UA produced optimal impacts.

## Figures and Tables

**Table 1:** Ingredients and nutrient levels of basic diet (as-fed basis, %).

Ingredient	Content	Nutritional Level ^(2)^	Content
Corn	15.00	Digestible energy/(MJ/kg)	10.06
Wheat bran	15.50	Dry matter	89.71
Dried whey	3.00	Crude protein	15.62
Soybean meal	15.00	Crude fiber	19.98
Peanut vine	8.00	Ether extract	2.91
Peanut shell	20.00	Neutral detergent fiber	35.70
Corn germ meal	3.00	Acid detergent fiber	17.98
Soybean oil	1.00	Calcium	1.26
Rice husk	4.00	Total phosphorus	0.62
Chili stalk powder	12.00		
Limestone	1.00		
CaHPO_4_	0.50		
NaCl	0.50		
L-Lys	0.35		
DL-Met	0.15		
Premix ^(1)^	1.00		
Total	100.00		

^(1)^ The premix provided the following per kg of the diet: Fe 70 mg, Cu 20 mg, Zn 70 mg, Mn 10 mg, Co 0.15 mg, I 0.2 mg, Se 0.25 mg, VE 50 mg, VK 2 mg, VB_1_ 2 mg, VB_2_ 6 mg, VB_3_ 50 mg, VB_5_ 50 mg, VB_6_ 2 mg, VB_12_ 0.02 mg, niacin 20 mg, pantothenic acid 12.5 mg, VA 10,000 IU, VD 900 IU, choline 1 000 mg, biotin 0.2 mg. ^(2)^ Digestible energy was calculated, while the others were measured.

**Table 2:** Sequence of primers for real-time PCR.

Target Gene	Accession No.	Primer Sequence (5′→3′)	Product Size (bp)
*TNF-α*	NM_001082263.1	F: GACGGGCTGTACCTCATCTACTC R: ACGGCGAAGCGGCTGAC	95
*IL-1β*	NM_001082201.1	F: TGTCCAGACGAGGGCATCCAG R: GAGCCACAACGACTGACAAGACC	85
*IL-6*	NM_001082064.2	F: GAGGCACTGGCGGAAGTCAATC R: TCAGCAGGCAGGTCTCATTATTCAC	94
*IL-8*	NM_001082293.1	F: GCTGTGGCTCTCTTGGCAACC R: ATTTGGGATGGAAAGGTGTGGAGTG	127
*IL-10*	NC_067389.1	F: AAACAAGAGCAAGGCAGTGG R: GGATGGAGTTCTCCTGGCTT	170
*Keap1*	XM_008251550.3	F: TCCTCAACCGCCTGCTCTATGC R: TCATCCGCCACTCGTTCCTCTC	99
*Nrf2*	MK645905.1	F: AAGCAACTCAGCACCTTGTATCTGG R: GAATACATTGCCGTCCCTCGTCTG	114
*HO-1*	XM_002711415.3	F: CCACCAAGTTCAAGCAGCTCTACC R: TTAGCCTCTTCCACCACCCTCTG	88
*NQO-1*	XM_002711667.3	F: CAGGAAGGACATCACAGGCAAGC R: CAGAATGGCAGGGACTCCAAACC	184
*SOD1*	NM_001082627.2	F: AAGGCTGTGTGCGTGCTGAAG R: GTCAGTCCTGTTATGCGTCCCTTG	107
*GSH-Px*	NM_001085444.1	F: CAGGAGAACGCCAAGAATGAGGAG R: GTTCACCTCGCACTTCTGGAAGAG	105
*GAPDH*	NM_001082253.1	F: CCACTTTGTGAAGCTCATTTCCT R: TCTCGTCCTCCTCTGGTGCT	142

**Table 3:** Effects of ursolic acid on growth performance of weaned rabbits in different groups.

Item	CON	UA Level, mg/kg			SEM	*p*-Value		
		50	100	200		Treatment	Linear	Quadratic
IBW, g	1023.18	1008.00	1033.25	1001.88	16.26	0.11	0.30	0.36
FBW, g	2178.32	2220.56	2198.66	2158.95	16.59	0.06	0.13	0.03
ADG, g	41.27 ^b^	43.31 ^a^	41.62 ^b^	41.32 ^b^	0.25	<0.01	0.32	0.06
ADFI, g	130.33 ^b^	135.14 ^a^	130.21 ^b^	130.74 ^ab^	0.66	0.02	0.38	0.21
F/G	3.16	3.12	3.13	3.16	0.02	0.76	0.77	0.38
Diarrhea rate, %	0.73	0.55	0.56	0.47	-	0.86	-	-
Mortality rate, %	2.50	2.50	5.00	5.00	-	0.88	-	-

^a,b^ Within a row, means with different superscript letters differ significantly (*p* < 0.05). IBW, initial body weight; FBW, final body weight; ADG, average daily gain; ADFI, average daily feed intake; F/G, feed to gain ratio.

**Table 4:** Effects of ursolic acid on organ indices of weaned rabbits.

Item	CON	UA Level, mg/kg			SEM	*p*-Value		
		50	100	200		Treatment	Linear	Quadratic
Liver index	2.89	2.89	2.86	2.99	0.05	0.86	0.52	0.60
Kidney index	0.62	0.62	0.63	0.62	0.01	0.90	0.58	0.77
Spleen index	0.05	0.06	0.07	0.06	0.01	0.23	0.44	0.06

**Table 5:** Effects of ursolic acid on ileal morphology of weaned rabbits.

Item	CON	UA Level, mg/kg			SEM	*p*-Value		
		50	100	200		Treatment	Linear	Quadratic
Villus height, μm	626.00 ^b^	788.73 ^a^	766.35 ^a^	775.36 ^a^	20.31	<0.01	<0.01	<0.01
Crypt depth, μm	128.44 ^a^	106.77 ^b^	113.79 ^ab^	112.54 ^ab^	2.95	0.03	0.09	0.04
Villus height/crypt depth	4.88 ^b^	7.40 ^a^	6.77 ^a^	6.89 ^a^	0.30	<0.01	<0.01	<0.01

^a,b^ Within a row, means with different superscript letters differ significantly (*p* < 0.05).

**Table 6:** Effects of ursolic acid on serum and cecal immune capacity of weaned rabbits.

Item	CON	UA Level, mg/kg			SEM	*p*-Value		
		50	100	200		Treatment	Linear	Quadratic
**Serum, μg/mL**								
IgM	1161.91	1170.38	1185.16	1184.36	18.91	0.97	0.68	0.83
IgG	3303.49	3421.67	3618.64	3539.85	64.54	0.35	0.18	0.27
IgA	5100.36	5117.62	5215.24	5321.79	67.77	0.67	0.23	0.95
C3	232.26	240.70	231.05	237.58	3.26	0.72	0.79	0.98
C4	227.40	245.60	246.62	240.79	2.51	0.05	0.08	0.03
**Cecum, pg/mL**								
sIgA	471.16 ^b^	561.33 ^a^	517.64 ^ab^	482.73 ^b^	10.11	<0.01	0.51	<0.01

^a,b^ Within a row, means with different superscript letters differ significantly (*p* < 0.05). IgM, immunoglobulin M; IgG, immunoglobulin G; IgA, immunoglobulin A; C3, complement 3; C4, complement 4; sIgA, secretory immunoglobulin A.

**Table 7:** Effects of ursolic acid on serum and cecal antioxidant capacity of weaned rabbits.

		UA Level, mg/kg			SEM	*p*-Value		
Item	CON	50	100	200		Treatment	Linear	Quadratic
**Serum**								
T-AOC, U/mL	39.56	41.75	39.31	39.35	0.61	0.44	0.55	0.61
GSH-PX, U/L	674.06	699.71	671.59	719.13	7.81	0.09	0.07	0.43
SOD, U/mL	333.78	347.37	353.14	353.59	3.57	0.17	0.06	0.20
CAT, U/mL	105.08 ^b^	110.02 ^ab^	110.75 ^ab^	114.23 ^a^	1.22	0.05	0.01	0.48
MDA, nmol/L	14.18	13.77	14.58	14.53	0.19	0.40	0.29	0.97
**Cecum**								
T-AOC, U/mg	4.06	4.55	3.99	3.75	0.13	0.21	0.17	0.37
GSH-PX, U/g	58.76	72.73	75.81	65.13	3.67	0.37	0.71	0.09
SOD, U/mg	27.37	33.90	24.63	28.21	1.50	0.11	0.22	0.67
CAT, U/mg	10.32 ^b^	14.69 ^a^	11.62 ^ab^	11.92 ^ab^	0.55	0.03	0.81	0.11
MDA, nmol/g	1.40	1.12	1.28	1.09	0.06	0.19	0.13	0.68

^a,b^ Within a row, means with different superscript letters differ significantly (*p* < 0.05). T-AOC, total antioxidant capacity; GSH-Px, glutathione peroxidase; SOD, superoxide dismutase; CAT, catalase; MDA, malondialdehyde.

**Table 8:** Effects of ursolic acid on serum and cecal inflammatory cytokines of weaned rabbits.

		UA Level, mg/kg			SEM	*p*-Value		
Item	CON	50	100	200		Treatment	Linear	Quadratic
**Serum, pg/mL**								
TNF-α	348.49 ^a^	267.60 ^b^	267.38 ^b^	275.88 ^b^	7.99	<0.01	<0.01	<0.01
IL-1β	85.61	84.35	89.19	91.65	1.65	0.40	0.43	0.38
IL-6	48.91	48.15	47.00	47.69	0.53	0.67	0.13	0.87
IL-8	171.04 ^a^	147.00 ^b^	146.62 ^b^	130.32 ^b^	3.65	<0.01	<0.01	0.12
**Cecum, pg/mg**								
TNF-α	65.60	64.97	63.52	61.13	0.88	0.30	0.06	0.88
IL-1β	17.40 ^a^	17.10 ^a^	15.90 ^ab^	15.18 ^b^	0.27	<0.01	<0.01	0.69
IL-6	28.49	28.02	28.43	27.11	0.30	0.36	0.13	0.55
IL-8	29.33	28.95	28.89	30.20	0.47	0.72	0.42	0.42
IL-10	13.33 ^b^	15.42 ^a^	15.21 ^ab^	14.69 ^ab^	0.29	0.03	0.20	0.01

^a,b^ Within a row, means with different superscript letters differ significantly (*p* < 0.05). TNF-α, tumor necrosis factor-α; IL-1β, interleukin-1β; IL-6, interleukin-6; IL-8, interleukin-8; IL-10, interleukin-10.

## Data Availability

The data associated with this study were not archived in a formal repository but can be obtained from the corresponding author upon reasonable request via e-mail.
